# Outcomes and risk of subsequent breast events in breast‐conserving surgery patients with BRCA1 and BRCA2 mutation

**DOI:** 10.1002/cam4.2836

**Published:** 2020-01-07

**Authors:** Fugui Ye, Liang Huang, Guantian Lang, Xin Hu, Genhong Di, Zhimin Shao, Ayong Cao

**Affiliations:** ^1^ Key Laboratory of Breast Cancer in Shanghai Department of Breast Surgery Fudan University Shanghai Cancer Center Shanghai China; ^2^ Department of Oncology Shanghai Medical College Fudan University Shanghai China; ^3^ Institutes of Biomedical Sciences Fudan University Shanghai China

**Keywords:** *BRCA1/2* mutation, breast cancer, breast‐conserving surgery, outcomes, subsequent breast events

## Abstract

**Purpose:**

Previous studies provide inconsistent interpretations of the effect of inherited genetic factors on the survival and prognosis of patients with breast cancer. The aim of this study was to examine the effect of germline *BRCA1* and *BRCA2* mutation on survival and subsequent breast events in Chinese women who underwent breast‐conserving surgery.

**Methods:**

A retrospective review of the clinical and pathological records was performed in patients diagnosed with primary invasive breast cancer between 2005 and 2018 in the cancer registry database. Clinicopathological data and data regarding treatment and outcomes, including date and site of disease progression, were collected. The survival outcomes and independent risk factors were conducted using SPSS.

**Results:**

Overall, a total of 501 patients who underwent breast‐conserving surgery were identified and subjected to analyses, of which 63 cases with *BRCA1* or *BRCA2* mutation. The median age at diagnosis was 41 (range, 24‐74) for carriers and 37 (range, 17‐84) for noncarriers. After a median follow‐up time of 61 months (range, 8‐161) and 70 months (range, 0‐153), respectively, in carriers and noncarriers, the overall survival (*P* = .173) and disease‐free survival (*P* = .424) were not significantly different. Analogously, there was no significant difference between the two groups about the outcomes of ipsilateral breast tumor recurrence (*P* = .348), yet the contralateral breast cancer (CBC) was overt worse than noncarriers (*P* < .001). When adjusted to confounding factors, *BRCA* mutation was the only independent risk factors to CBC (HR = 7.89, *P* = .01).

**Conclusion:**

In this study, *BRCA* mutation carriers have higher risk of CBC. And, *BRCA* mutation is the only independent risk factor to CBC. Therefore, intensive surveillance and follow‐up as well as more effective individual prevention are urgent. Decisions on alternatively effective prevention, especially the prevention of CBC, are urgent and should take into account patient prognosis and preferences.

## Graphical abstract

In our retrospective study, we conclude that breast‐conserving surgery is a reasonably safe and effective option for patients carrying a BRCA1 or BRCA2 mutation in China. BRCA mutation carriers have higher risk of CBC, and is the only independent risk factor to CBC. Decisions on alternatively effective prevention are urgent and should take into account patient prognosis and preferences.

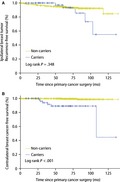

## 1 INTRODUCTION

Breast cancer is the most common noncutaneous malignancy of women worldwide.^1^ It is estimated that breast cancer in China alone accounts for 12.2% of global cases and 9.6% of related deaths.^2^ Nowadays, breast cancer is well acknowledged by heterogeneity, which in turn contributes to research complexity and treatment failure. Although the etiology and nature course of breast cancer remain largely to be elucidated, some inherent predisposing genes have been identified. *BRCA1* and *BRCA2* are the most characterized breast cancer susceptibility genes, and women carrying a germline mutation in *BRCA1/2* have an estimated 70% and 20%‐40% risk of developing breast cancer and ovarian cancer during their lifetime, respectively.^3^, ^4^ Studies indicate that breast cancer with an inherit deleterious germline *BRCA1/2* mutation, not only face a high risk of developing an ipsilateral^5^ or contralateral breast cancer,^6^ but also encounter an elevated risk of developing ovarian cancer.^7^ Therefore, intensively preclinical and clinical researches are conducted in this field.

Literatures demonstrate that *BRCA1*‐associated breast tumors show an aggressively pathological phenotype and are inclined to basal‐like subtype, while *BRCA2*‐associated breast cancers histologically and immunophenotypically tend to sporadic cancers and are predominantly luminal A‐like subtype.^8^, ^9^, ^10^, ^11^, ^12^, ^13^, ^14^ Currently, apart from clinical breast examination, mammography, ultrasound, and breast magnetic resonance imaging, it is wide recognition that prophylactic removal of the ovaries and risk‐reducing bilateral mastectomy are the most effective prevention measures to *BRCA*‐carriers and *BRCA*‐associated breast cancers.^15^, ^16^, ^17^, ^18^ Although the role of prophylactic oophorectomy is mixed in prior studies, it is suggested that prophylactic oophorectomy might not impact breast cancer incidence, but is associated with a favorable survival for this high‐risk population.^19^ Likewise, bilateral prophylactic mastectomy, sacrificing the quality of life, is regarded as one of the most effective measures to prevent *BRCA*‐associated breast cancer. Nevertheless, many *BRCA* carriers do not opt for this treatment and seek alternative preventive measures.^20^, ^21^

The subsequent breast events are the most important factors to tailoring the individually therapeutic strategy. It is suggested that there was no significant difference between *BRCA* carriers and noncarriers following breast‐conserving therapy as to the incidence of ipsilateral breast cancer recurrence. Conversely, published studies reported an increased incidence of contralateral breast cancer in mutation carriers, as compared with noncarriers.^22^, ^23^, ^24^, ^25^ Nevertheless, even with these preventive strategies, it is apparent that breast‐conserving therapy for *BRCA* carriers is not satisfactory, with a persistent high risk of developing contralateral breast cancer. It is worth noting that the conclusion that higher risk of subsequent breast events after breast‐conserving surgery in *BRCA* carriers is often derived from retrospective studies that have some intrinsic limitations.

Given the dilemmas, we conducted this single‐institutional retrospective study using propensity score matching method, with an effort to provide an almost accurate information of the outcomes and subsequent breast events after breast‐conserving surgery in *BRCA* carriers in Chinese women. And furthermore, to give more evidence‐based medicine concerning the practice of breast‐conserving surgery in *BRCA* carriers.

## 2 MATERIALS AND METHODS

### 2.1 Data collection and ethical statement

A retrospective review was conducted to identify breast cancer patients who underwent surgery at the Fudan University Shanghai Cancer Center between April 2005 and May 2018. The following variants were collected: genetic data (*BRCA* genetic test results), clinicopathological data (age at diagnosis, menopausal status, histopathology, unclear grade, tumor size, lymph node involvement, and hormone receptors status), and treatment data (surgical type and adjuvant systemic therapy according to local protocols). Patients with a previous invasive breast cancer or bilateral breast cancer were excluded. This study was approved by the Ethical Committee of the Shanghai Cancer Center of Fudan University.

### 2.2 *BRCA1/2* mutation test

Briefly, genomic DNA extracted from peripheral blood was subjected to NGS (next‐generation sequencing) according to the manufacture's instruction. All mutations considered disease‐associated were confirmed through Sanger sequencing. The details of procedures of NGS and interpretation of the mutation were described in our previous study.^26^

### 2.3 Definition of terms

Overall survival (OS) defined as the time from surgery to death from any cause. Disease‐free survival (DFS) defined as the time from surgery to any recurrence, distant metastasis, and death. Ipsilateral breast tumor recurrence (IPBT) defined as the interval from surgery to the reemergence of tumor in the previous affected breast or last follow‐up. Contralateral breast cancer (CBC) defined as the interval from surgery to the emergence of tumor in the contralateral breast.

### 2.4 Statistical analysis

Categorical variables were compared using the Pearson's Chi‐squared test or Fisher's exact test and independent *t*‐test for continuous variables. Kaplan‐Meier curves with corresponding log‐rank tests were conducted for survival curves and compared survival outcomes between different conditions of patient. Hazard ratios (HRs) and 95% confidential intervals (CIs) for univariable and multivariable analyses were calculated using Cox proportional‐hazards models. All tests were two‐sided, and *P* < .05 was considered statistically significant. Statistical analysis was performed by SPSS for windows (version 23.0, SPSS Inc).

## 3 RESULTS

### 3.1 Basic characteristics of study cohort

Through retrospective review, we identified 501 patients who underwent breast‐conserving surgery, with 63 *BRCA1/2* mutation carriers and 438 noncarriers with invasive breast cancer in this study. The demographic and clinicopathological features by group are shown in Table 1. Median age at diagnosis was 41 years (range, 24‐74) for carriers and 37 years (range, 17‐84) for noncarriers. The majority of participants were premenopausal with 65.08% in carriers and 72.15% in noncarriers, respectively. As expected, *BRCA1/2* mutation carriers showed more aggressive behaviors, including with significantly more grade 3 (*P* < .001) and more hormone receptor negative (*P* < .001), as well as higher proliferation index (*P* = .039) patients as compare with noncarriers. Besides, *BRCA*‐associated tumors tend to be HER‐2‐negative compared with noncarriers (95.24% vs 81.96%). However, we failed to conclude any statistically significant with respect to tumor size, histological type, and lymph node involvement. Median follow‐up time was 61 months (range, 8‐161) and 70 months (range, 0‐153), respectively, in carriers and noncarriers.

**Table 1:** Patient and tumor characteristics of *BRCA1/2* mutation carriers and controls

Characteristic	Carriers (n = 63)		Noncarriers (n = 438)		*P* value
	No.	%	No.	%	
Age at diagnosis (y)					
Median	41		37		
Range	33‐47		32‐50		
Menopausal status					
Premenopausal	41	65.08	316	72.15	.247
Postmenopausal	22	34.92	122	27.85	
Tumor size (mm)					
pT1	34	53.97	276	63.01	.182
pT2	22	34.92	107	24.43	
pT3	0	0.00	4	0.91	
Unknown	7	11.11	51	11.64	
Lymph node status					
Negative	48	76.19	304	69.41	.333
Positive	14	22.22	121	27.63	
Unknown	1	1.59	13	2.97	
Histology					
IDC	57	90.48	377	86.07	.337
Others	6	9.52	61	13.93	
Nuclear grade					
I and II	12	19.05	189	43.15	**<.001**
III	40	63.49	158	36.07	
Unknown	11	17.46	91	20.78	
Estrogen receptor					
Negative	49	77.78	164	37.44	**<.001**
Positive	14	22.22	273	62.33	
Progesterone receptor					
Negative	49	77.78	180	41.10	**<.001**
Positive	14	22.22	257	58.68	
HER‐2/neu					
Negative	60	95.24	359	81.96	**.008**
Overexpressed	3	4.76	78	17.81	
Ki‐67 (%)					
<14	4	6.35	77	17.58	**.016**
≥14	50	79.37	283	64.61	
Unknown	9	14.29	78	17.81	

The bold values indicate that BRCA‐carriers tend to be more grade 3, higher proportion of triple negative breast cancer patients and Ki‐67.

Abbreviations: HER‐2, human epidermal growth factor receptor‐2; IDC, infiltrating ductal carcinoma; Ki‐67, cell proliferation index; Nuclear grade I, well differentiation; Nuclear grade II, moderate differentiation; Nuclear grade III, poor differentiation; pT1, pathological tumor size ≤2 cm; pT2, 2 cm<pathological tumor size ≤5 cm; pT3, pathological tumor size >5 cm.

### 3.2 Oncologic outcomes

**Figure 1:**
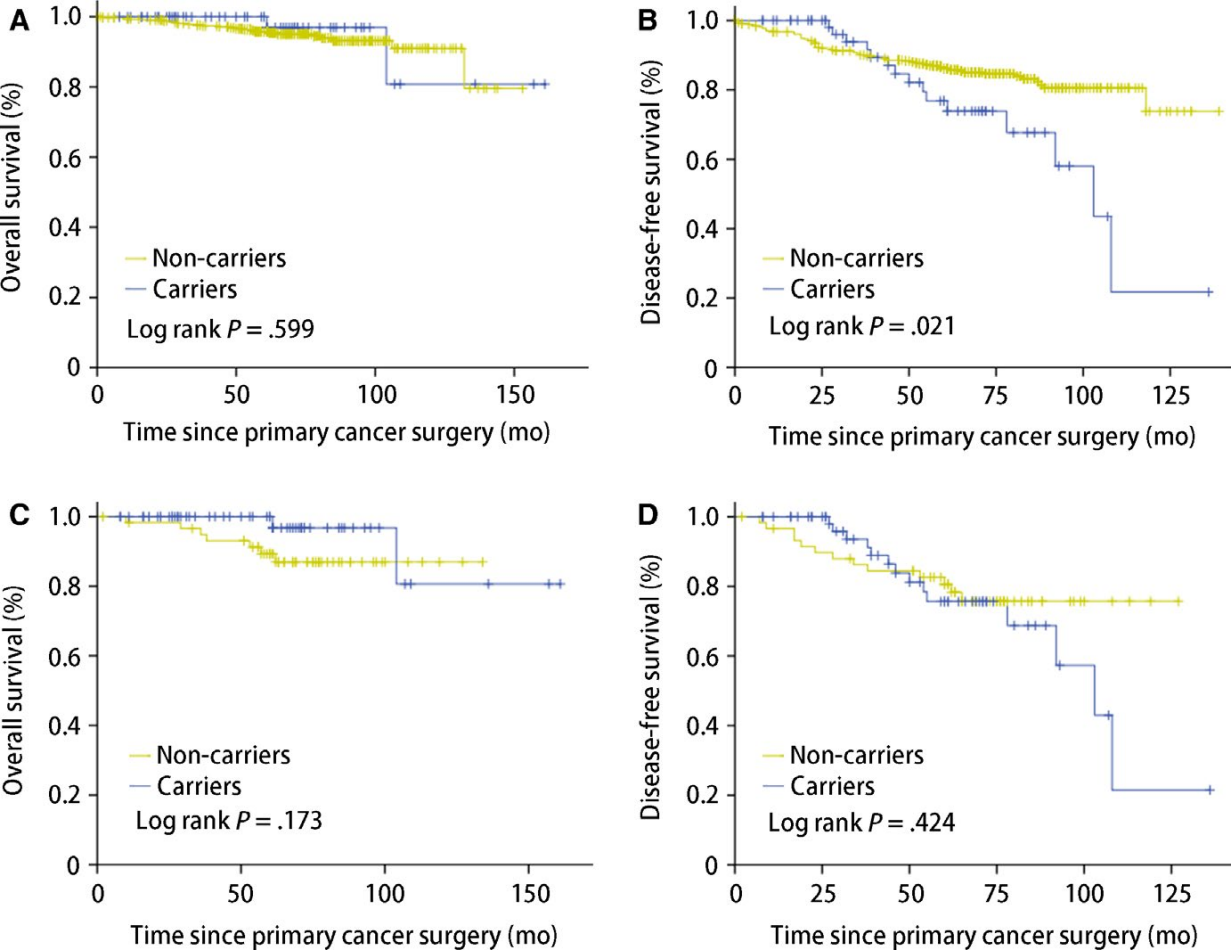
Survival outcomes after breast cancer‐conserving surgery by *BRCA1/2* mutation status estimated by univariate. A, Overall survival, (B) Disease‐free survival, (C) Adjusted overall survival, (D) Adjusted disease‐free survival

**Figure 2:**
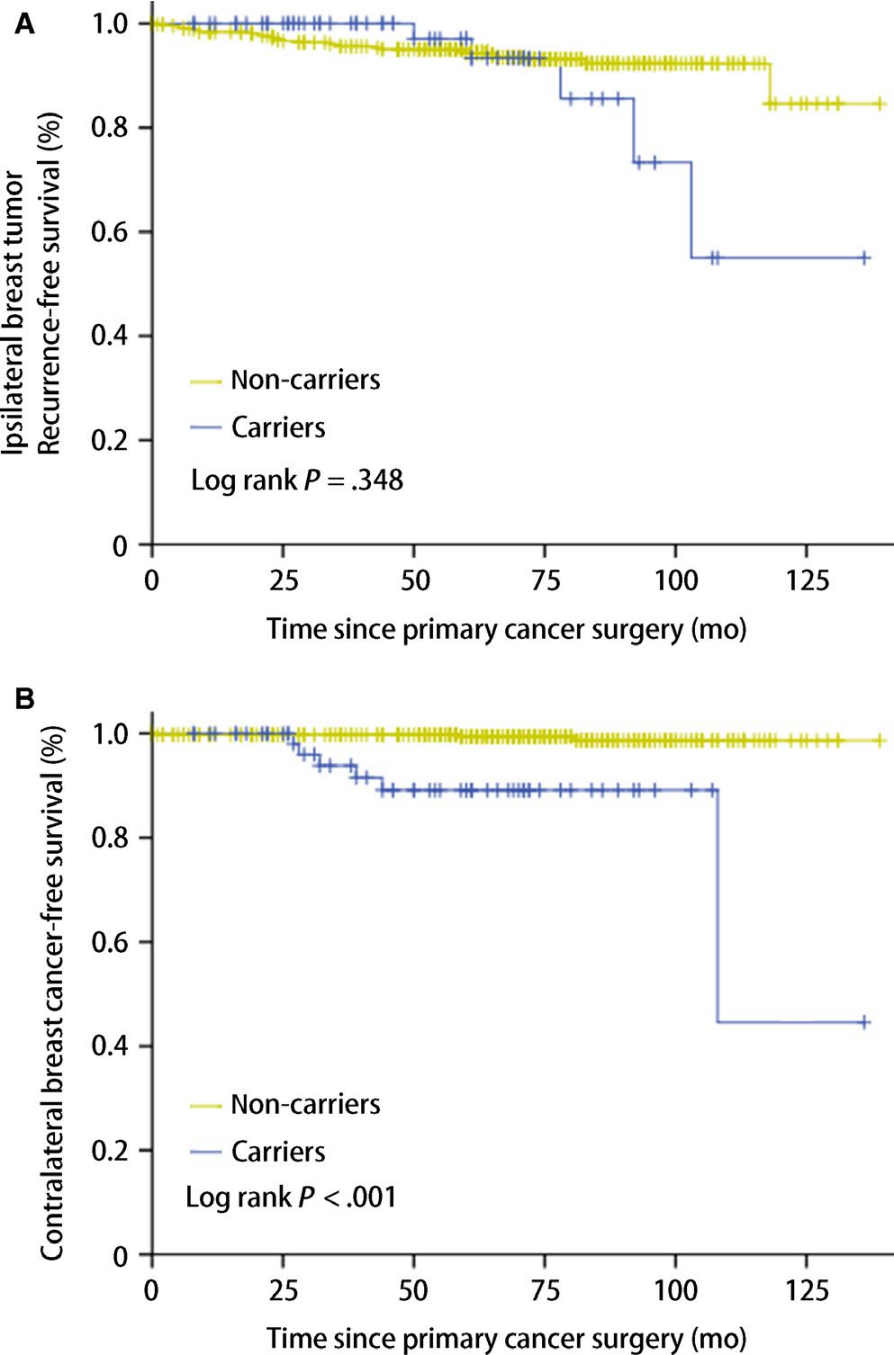
Subsequent breast events according to *BRCA1/2* mutation status estimated by univariate. (A) Ipsilateral breast tumor recurrence‐free survival, (B) Contralateral breast cancer‐free survival

In the *BRCA* mutation carriers, there were 15 (23.81%) first events, including five ipsilateral breast tumor, six contralateral breast cancer, three distant metastases, and one other primary tumor. Whereas in the noncarriers, 63 (14.38%) first events were observed, with 27 ipsilateral breast tumor, three contralateral breast cancer, three regional recurrence, 25 distant metastases, and five other primary tumors (Table 2). There was no significant difference in OS between the two groups (*P* = .599; Figure 1A), even adjusted to tumor size, lymph node status, hormone receptor status, Ki 67, and treatment schedule (*P* = .173; Figure 1C). Of note, *BRCA* mutation carriers showed worse DFS (*P* = .021; Figure 1B), but was comparable to noncarriers when adjusted to the mentioned confounding factors (*P* = .424; Figure 1D). The outcomes of IPBT were comparable (*P* = .348), yet the CBC was overt worse than noncarriers (*P* < .001; Figure 2).

**Table 2:** Frequency of events

Description of events	Carriers (n = 63)		Noncarriers (n = 438)	
	No.	%	No.	%
First events	15	23.81	63	14.38
Ipsilateral breast tumor	5	7.94	27	6.16
Regional	0	0.00	3	0.68
Distant	3	4.76	25	5.71
Contralateral breast cancer	6	9.52	3	0.68
Other primary tumor	1	1.59	5	1.14
Death (any cause)	2	3.17	24	5.48

### 3.3 Risk factors of CBC

**Figure 3:**
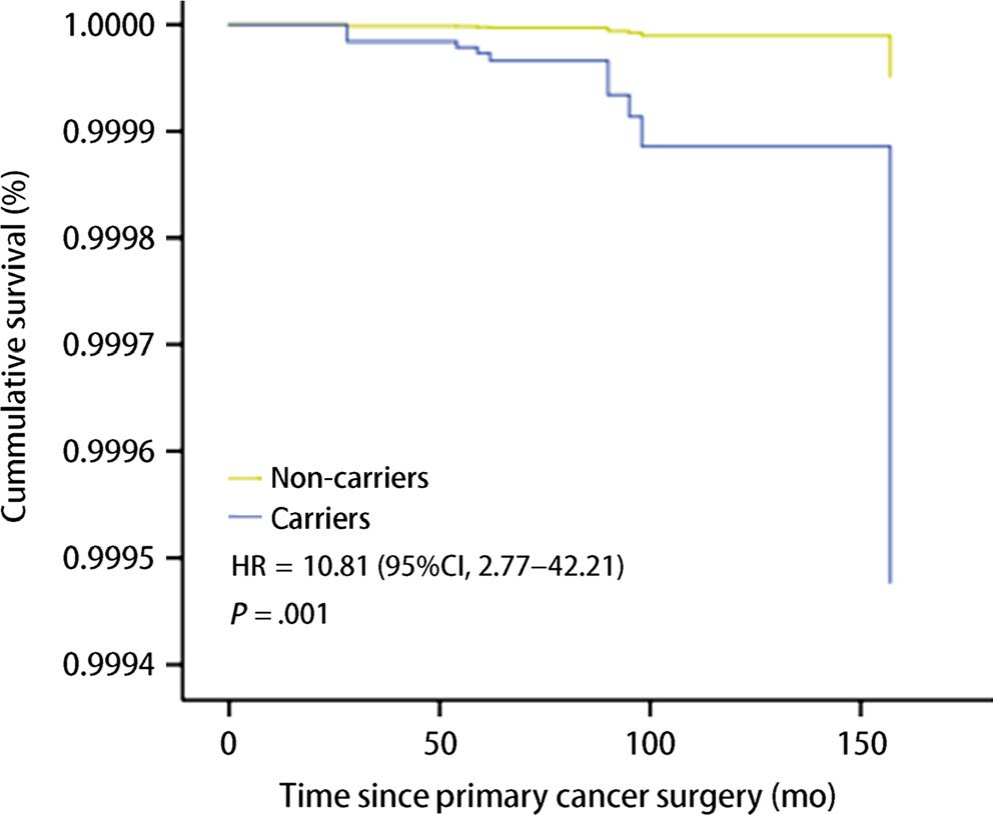
Contralateral breast cancer‐free survival after breast‐conserving surgery estimated by BRCA1/2 mutation status in multivariate analysis

The relationship of emergence of CBC with clinicopathological factors was evaluated by Cox proportional‐hazards model. The univariable and multivariable HRs associated with each of the factors are presented in Table 3. CBC was prone to occur in *BRCA* mutation carriers as compared to noncarriers (HR = 15.76, *P* < .001). Beyond that, postmenopausal status suggested a borderline significant increase in the risk of CBC (HR = 3.99, *P* = .06). However, only *BRCA1/2* mutation status was an independent risk factor to CBC in multivariate analysis (HR = 10.81, *P* = .01). Figure 3 demonstrated the probability of CBC in the light of *BRCA1/2* mutation status.

**Table 3:** Risk of CBC associated with clinicopathological characteristics

Characteristic	Univariate			Multivariate		
	Hazard ratio	95% CI	*P*	Hazard ratio	95% CI	*P*
BRCA1/2 status						
Noncarriers	1(ref)			1(ref)		
Carriers	16.63	4.86‐56.92	**<.001**	10.81	2.77‐42.21	**.001**
Age at diagnosis (years)						
≤40	1(ref)			1(ref)		
>40	0.85	0.28‐2.60	.77	0.46	0.12‐1.72	.25
Menopausal status						
Postmenopausal	1(ref)			1(ref)		
Premenopausal	0.25	0.08‐0.82	**.02**	0.45	0.12‐1.70	.24
Tumor size (mm)						
≤20	1(ref)			1(ref)		
>20	0.98	0.26‐3.69	.97	0.77	0.17‐3.54	.74
Lymph node status						
Negative	1(ref)			1(ref)		
Positive	0.88	0.27‐2.92	.84	1.24	0.28‐5.51	.78
Nuclear grade						
I and II	1(ref)			1(ref)		
III	88 068.47	0‐∞	.9	71 849.61	0‐∞	.92
Estrogen receptors						
Negative	1(ref)			1(ref)		
Positive	0.6	0.2‐1.79	.36	1.62	0.21‐12.68	.65
Progesterone receptors						
Negative	1(ref)			1(ref)		
Positive	0.62	0.21‐1.86	.39	1.15	0.15‐9.04	.9
HER‐2/neu						
Negative	1(ref)			1(ref)		
Overexpressed	0.04	0.00‐29.15	.33	<0.001	0‐∞	.96
Ki‐67						
<14	1(ref)			1(ref)		
≥14	0.38	0.08‐1.83	.23	0.26	0.03‐2.43	.24

The bold values derived from univariated analysis indicate that BRCA‐mutation status and menopausal status might attribute to risk of contralateral breast cancer (CBC), however, only BRCA‐mutation status confer to the independent risk factor of CBC.

Abbreviations: CBC, contralateral breast cancer; HER‐2, human epidermal growth factor receptor‐2; Ki‐67, cell proliferation index; Nuclear grade I, well differentiation; Nuclear grade II, moderate differentiation; Nuclear grade III, poor differentiation.

## 4 DISCUSSION

This retrospective study indicated no significant difference in OS between patients carrying a *BRCA1* or *BRCA2* mutation and sporadic breast cancers. This result did not vary even when using the propensity score matching method. Inversely, DFS was dramatically worse in *BRCA1/2* mutation carriers. Even if *BRCA1/2*‐associated cases were deemed as an aggressive subtype, however, DFS was no longer lethal when adjusted to clinicopathological and therapeutic confounding factors. Our results were in broad agreement with more recent studies, although others had reported conflicting results.^27^, ^28^, ^29^, ^30^, ^31^, ^32^

The percentage of *BRCA*‐associated patients in our study (12.6%) was similar to a prior prospective cohort study,^33^ higher than that reported in clinic‐based screening (9.1%).^26^ Besides, unlike some studies illustrated that *BRCA*‐associated patients tend to have a greater likelihood of higher nuclear grade and lymph node involvement, no statistically significant difference was presented in the current study. One theory that could explain the discrepancies was that the next‐generation sequencing and advances in the mammographic screening promote the easier access of genetic testing and earlier detecting of breast cancer. Besides, differences in ethnic background, recruitment criteria, and pathogenic mutation location may exert some effect. It was reported that the risk of breast cancer increased substantially between the ages of 30 and 50 years for *BRCA1* carriers, while the risk was highest between the ages of 40 and 60 years for *BRCA2* carriers,^19^ which was simulated to our results. Other aspects of basic characteristics of this study were favor of previous literatures.^34^, ^35^

The subsequent breast events are often the major concern and obstacle to decide a medical schedule for *BRCA*‐associated patients. Although the standard procedures of treatment for *BRCA* mutation carriers remain a matter of debate, increasing evidence supports that breast‐conserving surgery is a rational option for *BRCA* carriers. In this study, after breast‐conserving surgery, subsequent breast events occurred in 15 (23.81%) carriers and 63 (14.38%) noncarriers. In addition, distinct from IPBT, the incidence of CBC was apparently higher in carriers than noncarriers. These results were in line with the majority of published studies that derived the conclusion of no difference in local recurrence,^36^ whereas increased incidence of CBC was associated with *BRCA1/2* mutation.^4^, ^22^, ^37^, ^38^, ^39^, ^40^, ^41^ Till now, comprehensive research has been administrated to seek optimum prevention of CBC in *BRCA* mutation carriers.

Despite *BRCA1* and *BRCA2* mutations were identified a couple of decades and risk‐reducing mastectomy remains the gold standard, for fear of aesthetics, increasing mutation carriers have strong preferences for noninvasive prevention, it is timely that an effective breast cancer risk reduction option be identified.^19^ Bilateral salpingo‐oophorectomy, to some extent, is advocated for *BRCA* mutation carriers even divergent findings existed.^42^, ^43^, ^44^ Chemoprevention, with several agents, is also provided for *BRCA* mutation carriers, but the acceptability is rather low.^45^, ^46^, ^47^ Recently, E. Evron et.al suggested that the addition of contralateral breast irradiation to the routine local‐regional treatment was associated with a significant reduction of subsequent contralateral breast cancers and a delay in their onset.^18^ In light of efficacy and safety, not only the preliminary research, but also the clinical trials proposed prophylactic mammary irradiation for prevention of CBC in *BRCA* mutation setting.^22^, ^48^, ^49^, ^50^, ^51^, ^52^

Although great efforts were made to balance the patient characteristics and treatment modalities, limitations of this study mainly lay in the ascertainment biases introduced by retrospective study. Otherwise, small sample size and an inadequate follow‐up time probably interpret many discrepancies with previous studies. At the time of this report, the median follow‐up is relatively short. Studies suggested that the subsequent breast events are time‐dependent and possible to decrease over time.

In summary, our study confirmed higher risk of CBC for *BRCA* mutation carriers in majority of Chinese women. Consequently, *BRCA* carrier patients should be fully informed when choosing breast‐conserving surgery. Furthermore, intensive surveillance and follow‐up as well as more effective individual prevention are urgent. Clinical trials with large number size and long‐term follow‐up time are needed to confirm this conclusion.

## CONFLICT OF INTERESTS

All authors declare that they have no competing interests.

## Data Availability

None (for ethical reasons).
